# β2-and β3-Adrenergic Receptors Contribute to Cancer-Evoked Pain in a Mouse Model of Osteosarcoma *via* Modulation of Neural Macrophages

**DOI:** 10.3389/fphar.2021.697912

**Published:** 2021-09-27

**Authors:** Gennaro Bruno, Francesco De Logu, Daniel Souza Monteiro de Araujo, Angela Subbiani, Federica Lunardi, Sofia Rettori, Romina Nassini, Claudio Favre, Maura Calvani

**Affiliations:** ^1^ Department of Health Sciences, Clinical Pharmacology Unit, University of Florence, Florence, Italy; ^2^ Division of Pediatric Oncology/Hematology, Meyer University Children’s Hospital, Florence, Italy; ^3^ Department of Clinical and Experimental Medicine, University of Pisa, Pisa, Italy

**Keywords:** β-adrenergic receptors, cancer pain, osteosarcoma, neuroinflammation, macrophages

## Abstract

The mechanisms involved in the development and maintenance of cancer pain remain largely unidentified. Recently, it has been reported that β-adrenergic receptors (β-ARs), mainly β2-and β3-ARs, contribute to tumor proliferation and progression and may favor cancer-associated pain and neuroinflammation. However, the mechanism underlying β-ARs in cancer pain is still unknown. Here, we investigated the role of β1-, β2-and β3-ARs in a mouse model of cancer pain generated by the para-tibial injection of K7M2 osteosarcoma cells. Results showed a rapid tumor growth in the soft tissue associated with the development of mechanical allodynia in the hind paw ipsilateral to the injected site. In addition to reduce tumor growth, both propranolol and SR59230A, β1-/β2-and β3-AR antagonists, respectively, attenuated mechanical allodynia, the number of macrophages and an oxidative stress by-product accumulated in the ipsilateral tibial nerve. The selective β1-AR antagonist atenolol was able to slightly reduce the tumor growth but showed no effect in reducing the development of mechanical allodynia. Results suggest that the development of the mechanical allodynia in K7M2 osteosarcoma-bearing mice is mediated by oxidative stress associated with the recruitment of neural macrophages, and that antagonism of β2-and β3-ARs contribute not solely to the reduction of tumor growth, but also in cancer pain. Thus, the targeting of the β2-and β3-ARs signaling may be a promising therapeutic strategy against both tumor progression and the development of cancer-evoke pain in osteosarcoma.

## Introduction

Pain is one of the most common invalidating symptoms of cancer, affecting approximately 70% of cancer patients worldwide every year (van den Beuken-van Everdingen et al., 2007). Despite the significant advances in understanding, early detection, and treatment of cancer, progress in the knowledge of pain related to cancer and improvement in the use of analgesics have been limited. Causes of cancer pain are multifactorial and complex and are likely connected with an array of tumor- and host-related factors (Lam and Schmidt, 2010). Numerous receptors and their activators have been studied for a better understanding of the cancer pain mechanisms. These comprise the transient receptor potential (TRP) channels, including the vanilloid 1 (TRPV1) and ankyrin 1 (TRPA1) subtypes which are involved in bone (Ghilardi et al., 2005), and melanoma (Antoniazzi et al., 2019; De Logu et al., 2021) cancer pain models, respectively. The contribution of the acid-sensing ion channels (ASICs) has been reported in an osteolysis-induced bone cancer pain (Nagae et al., 2007) and the protease-activator receptor 2 (PAR2) in peripheral neurons is the target of serine proteases and tryptase released from cancer cells to promote the prolonged mechanical allodynia in mouse cancer nociceptive models (Lam and Schmidt, 2010).

β-ARs are G protein coupled receptors constituted of seven transmembrane domains (Hein and Kobilka, 1995; Hein, 2006), which mediate catecholamine-induced activation of adenylate cyclase. Three β-AR subtypes have been identified, the β1-, β2-and β3-ARs, which are mostly known for their role in the regulation of cardiovascular, airway, uterine, and other peripheral functions (Hein, 2006). β-ARs have also been implicated in functions of the central nervous system, including the regulation of sympathetic tone, as well as different disorders, including those associated with learning and memory, mood and food intake (Hein and Kobilka, 1995; Hein, 2006). Given their expression in peripheral nervous systems (Hein, 2006), emerging evidence also suggests a contribution of β-ARs in modulating different chronic pain conditions (Yalcin et al., 2010; Bunevicius et al., 2013; Zhang et al., 2018). β-ARs seem to be involved in pain processes mainly through activation of immunoregulatory cells, including T-cells (Laukova et al., 2012; Slota et al., 2015), mast cells (Chi et al., 2004), and macrophages (Chiarella et al., 2014; Kim et al., 2014) with associated increases in pro-inflammatory cytokines and nociceptors sensitization (Binshtok et al., 2008; Czeschik et al., 2008). Expression of β2-ARs within the nociceptive system suggested their potential implication in nociception and pain (Nicholson et al., 2005; Kanno et al., 2010; Hartung et al., 2014; Ciszek et al., 2016; Zhang et al., 2018), and human genetic studies also confirmed the contribution of β2-ARs to chronic pain disorders (Diatchenko et al., 2006). However, the recruitment of β2-ARs in the participation to pro- or antinociceptive mechanisms might depend on the considered stimulus since analgesic actions were observed with an agonist in a visceral pain model (Bentley and Starr, 1986) and with an antagonist in inflammatory pain models (Parada et al., 2003; Nackley et al., 2007). Conversely, either the stimulation or inhibition of the β1-AR subtype did not directly participate to the antinociceptive mechanisms in models of neuropathic pain (Yalcin et al., 2009a; Yalcin et al., 2009b). Instead, the β3-AR subtype has been primarily associated with metabolic regulation both in physiological and pathological conditions (Schena and Caplan, 2019).

Neuropathic pain resulting from nerve injury may be attributable to an excessive immune response leading to the development of neuroinflammation in the peripheral (Trevisan et al., 2016; De Logu et al., 2017) or central nervous system (Tsuda et al., 2003). Neuroinflammation is an established cause of neuropathic pain in the sciatic nerve ligation mouse model (De Logu et al., 2017), in the reperfusion ischemia mouse model (De Logu et al., 2020a), and in the trigeminal nerve ligation mouse model (Trevisan et al., 2016). Moreover, inflammatory processes are involved in different pathologies, including cancer. It is widely recognized that the immune system interacts with the sensory nervous system contributing to maintenance of chronic pain (De Logu et al., 2017; De Logu et al., 2020a).

In model of sciatic nerve injury in rat, noradrenergic perivascular axons germinate in the dorsal root ganglia (DRG) to form unusual structures around axotomized sensory neurons of large diameter able to mediate pain signaling (McLachlan et al., 1993). In addition, increased mRNA levels of β2-AR in ipsilateral DRGs after sciatic nerve injury has been observed (Maruo et al., 2006). Moreover, the neuropathy produced by the axotomy of DRG neurons was relieved by manipulation of adrenergic receptors (ARs) (Chung and Chung, 2002), and noradrenaline, by the β3-AR stimulation, has been reported to mediate the release of adenosine-triphosphate (ATP) from DRG neurons, thus causing mechanical allodynia in rat model of peripheral nerve injury (Kanno et al., 2010). Immune cells respond by activation of ARs, primarily the β2-ARs, which regulate a variety of functions ranging from cellular migration to cytokine secretion (Wieduwild et al., 2020). However, little is known about the involvement of β-ARs in cancer evoked-pain.

Here, we investigated whether β1-, β2-and β3-ARs contribute to pain in a mouse model of osteosarcoma generated by the para-tibial injection of K7M2 cells, using different β-ARs blockers. Although the selectivity of the most of β-ARs blockers is questionable, based on previously reported data (Dal Monte et al., 2013; Calvani et al., 2019) we used atenolol, propranolol and SR59230A as blockers to modulate β1-, β2-and β3-ARs downstream effects, respectively. We observed that pharmacological antagonism of β2-and β3-ARs, reduced tumor growth and the ensuing mechanical allodynia caused by K7M2 osteosarcoma cells inoculation. Tumor growth was associated with an increase in the number of F4/80^+^ cells inside the tibial nerve that was attenuated by β2-and β3-ARs antagonism. These results corroborate the role of neuroinflammation in cancer pain and introduce the role of β-ARs as crucial players in the development and maintenance of chronic pain and in the modulation of neuroinflammation.

## Materials and Methods

### Tumor Syngeneic Model

BALB/c mice (male, 4 weeks old, Envigo RMS) were used. Mice were housed in a temperature- and humidity-controlled vivarium (12 h dark/light cycle, free access to food and water). Behavioral experiments were done in a quiet, temperature-controlled (20–22°C) room between 9 am and 5 pm and were performed by an operator blinded to the drug treatment. Animals were anesthetized with a mixture of ketamine and xylazine (90 mg/kg and 3 mg/kg, respectively, i.p.) and euthanized with inhaled CO_2_ plus 10–50% O_2_.

Osteosarcoma K7M2 (CRL-2836, American Type Culture Collection, ATCC) murine cells were grown in Dulbecco′s Modified Eagle′s Medium (DMEM) supplemented with 10% fetal bovine serum (FBS), penicillin/streptomycin solution (1x) and glutamine (2 mM) at 37°C, 5% CO_2_ atmosphere, and 21% O_2_.

For cells inoculation, 50 µl of K7M2 (7 × 10^5^) cells were suspended in phosphate buffer saline (PBS) and injected para-tibial. Control groups (sham) were injected with 50 µl of PBS. K7M2 cell lines are isogenic with the BALB/c mouse strain. Tumor growth rate was evaluated by measuring tumor mass with a caliber, and tumor mass volume calculated as volume = [(length x width)2/2]. Mice were sacrificed at day 20 after K7M2 cells inoculation or sham. SR59230A (Tocris Bioscence), propranolol (Merck Life Science) and atenolol (Merck Life Science) (10 mg/kg i.p.) or vehicle (NaCl 0.9%) were administered twice a day (every 8 h) starting from day 10 after K7M2 cells inoculation, when a palpable tumor was present, or sham. Phenyl-alpha-tert-butyl nitrone (PBN, 100 mg/kg, i.p.) or vehicle (4% dimethyl sulfoxide, DMSO, 4% tween 80 in 0.9% NaCl) was given at day 14 after K7M2 cells inoculation.

### Mechanical Allodynia

The measurement of mechanical paw withdrawal threshold (PWT) was carried out using von Frey filaments of increasing stiffness (0.02–2 g) applied to the plantar surface of the mouse hindpaw, according to the up-and-down paradigm (Chaplan et al., 1994). The 50% mechanical paw-withdrawal threshold (g) response was then calculated from the resulting scores.

### Tibial Nerve Dissociation and Flow Cytometry Analysis

**FIGURE 1:**
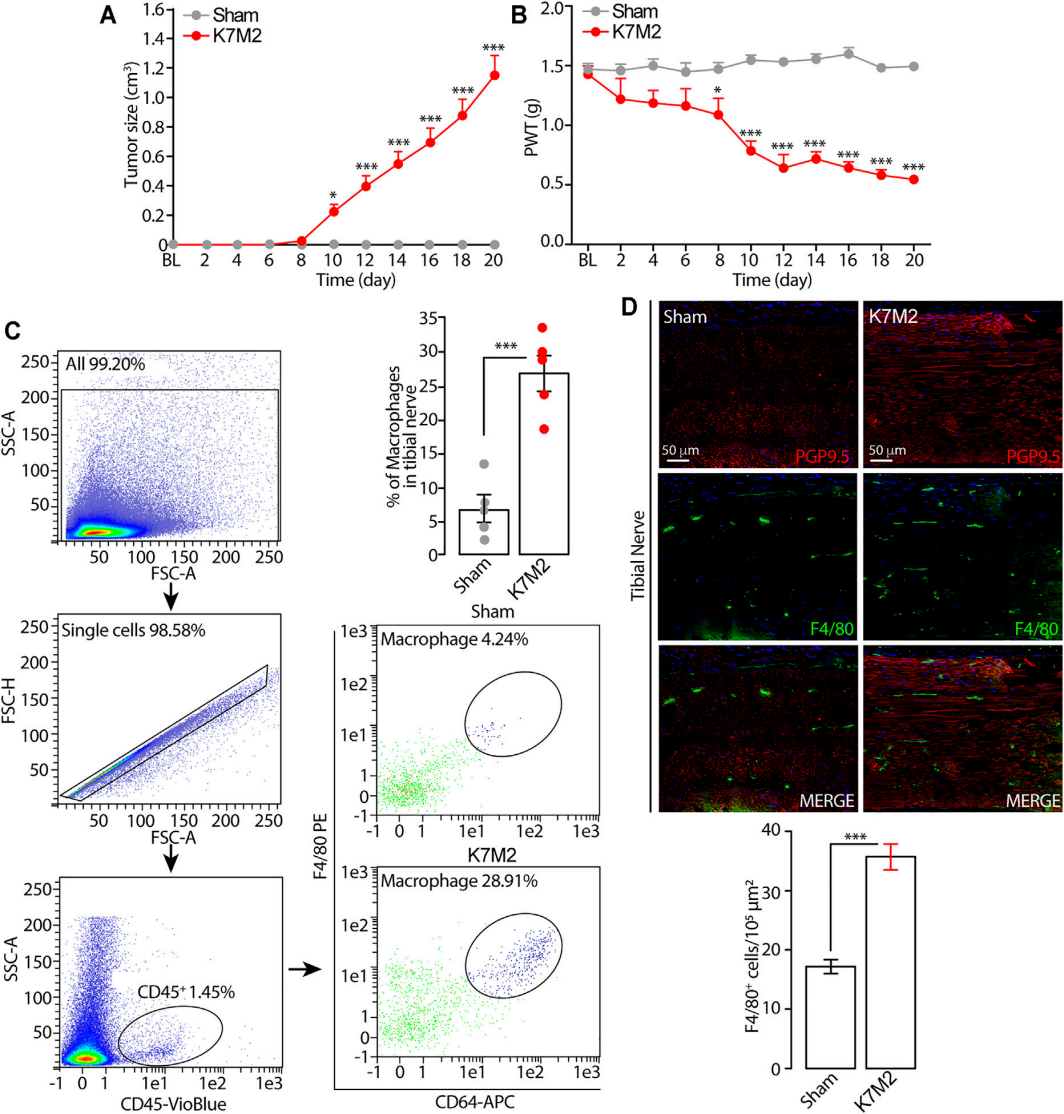
Tumor growth and mechanical allodynia induced by K7M2 osteosarcoma cells in BALB/c mice **(A** and **B)** Time dependent increase in tumor size and mechanical allodynia after para-tibial K7M2 osteosarcoma cell inoculation or sham in BALB/c mice **(C)** Representative gating strategy of flow cytometry analysis and relative quantification of F4/80^+^/CD64^+^ monocytes/macrophages in tibial nerve, and **(D)** typical images and pooled data of F4/80^+^ cells in the ipsilateral tibial nerve (stained with the neural marker PGP9.5) after para-tibial K7M2 osteosarcoma cell inoculation or sham in BALB/c mice. BL, baseline. PWT, paw withdrawal threshold. N = 6 mice. *p < 0.05, ***p < 0.001 K7M2 vs Sham. Data are presented as mean ± SEM, data points overlaid (C, D). Two-way ANOVA and Bonferroni post hoc test (A, B), unpaired student t-test (C, D).

Tibial nerves were dissected from euthanized mice and mechanically dissociated to obtain a single cell suspension. Briefly, nerves were dissociated in a solution containing HEPES (25 mM), Hanks’ Balanced Salt solution (HBSS, 1x), FBS 10% and dnase (10 µM) by using the gentleMACS Octo Dissociator (Miltenyi Biotec). Cells were then stained with anti-CD45-VioBlue conjugated antibody (130-110-664, Miltenyi Biotec), anti-F4/80-PE conjugated antibody (130-116-449, Miltenyi Biotec), and anti-CD64-APC conjugated antibody (130-126-950, Miltenyi Biotec). After staining, cells were subjected to flow cytometry by using a Miltenyi Biotec MACSQuant Analyzer 10. Results were analyzed by using FlowlogicTM Software. Gating strategy is reported in Figure 1A.

### Immunofluorescence

The tumor and tibial nerve were dissected from anesthetized and transcardially perfused with PBS, followed by 4% paraformaldehyde, mice. The tibial nerve and the hindlimb were postfixed for 24 h, and cryoprotected in sucrose 30%. Immunofluorescence staining was performed according to standard procedures. Briefly, after the antibody retrieval with citrate buffer pH 6, tissue sections (10 µm) were incubated with the following primary antibodies: F4/80 [1:50, MA516624, rat monoclonal (Cl:A3-1), RRID:AB_2538120 Thermo Fisher Scientific], PGP9.5 (1:250, ab108986, rabbit monoclonal, RRID:AB_ 10891773 Abcam), β1 receptor (1:400, ab3442, rabbit polyclonal, RRID:AB_ 10890808 Abcam), β2 receptor (1:100, ab182136, rabbit monoclonal, RRID:AB_ 2747383 Abcam), β3 receptor (1:100, ab94506, rabbit monoclonal, RRID:AB_ 10863818 Abcam) diluted in block solution (5% normal goat serum and normal donkey serum, in PBS and triton-x100, PBST) 1 h at room temperature. Sections were then incubated for 2 h in the dark with the fluorescent secondary antibody polyclonal, Alexa Fluor^®^ 488 (1:600, Thermo Fisher Scientific) or Alexa Fluor^®^ 594 (1:600, Thermo Fisher Scientific). Sections were coverslipped using a water-based mounting medium with 4′6′-diamidino-2-phenylindole (DAPI, Abcam). The analysis of negative controls (non-immune serum) was simultaneously performed to exclude the presence of non-specific immunofluorescent staining, cross-immunostaining, or fluorescence bleed-through. For histological evaluation, sections were stained with hematoxylin/eosin and based on the morphology, the boundaries of the nerve trunk corresponding to the epineurium were identified and reported in adjacent immunofluorescence images with dashed lines. The number of F4/80^+^ cells was counted in 10^4^ μm^2^ boxes in the tibial nerve trunk.

### H_2_O_2_ Assay

H_2_O_2_ level was assessed by using the Amplex Red^®^ assay (Thermo Fisher Scientific). Tibial nerve tissue was rapidly removed and placed into modified Krebs/HEPES buffer [composition in mmol/l: 99.01 NaCl, 4.69 KCl, 2.50 CaCl_2_, 1.20 MgSO_4_, 1.03 KH_2_PO_4_, 25.0 NaHCO_3_, 20.0 Na-HEPES, and 5.6 glucose (pH 7.4)]. Samples were minced and incubated with Amplex red (100 μM) and HRP (1 U/ml) (1 h, 37°C) in modified Krebs/HEPES buffer protected from light. Fluorescence excitation and emission were at 540 and 590 nm, respectively. H_2_O_2_ production was calculated using H_2_O_2_ standard and expressed as μmol/l of mg of dry tissue.

RAW 264.7 cells were seeded in 96-well plates (30.000 cells/well) and grown in phenol red-free Roswell Park Memorial Institute (RPMI). The cultured medium was replaced with Krebs-Ringer phosphate (KRP, composition in mM: 2 CaCl_2_; 5.4 KCl; 0.4 MgSO_4_; 135 NaCl; 10 d-glucose; 10 HEPES [pH 7.4]) added with SR59230A, propranolol, atenolol (all, 100 nM) or vehicle (0.01% DMSO in KRP) for 20 min at room temperature. Cells were activated by the addition of phorbol-myristate-acetate (PMA, 16 μM), along with Amplex Red (50 μM) and HRP (1 U/ml) at a total volume of 100 μl. Signal was detected 60 min after exposure to the stimuli, at 560 nm H_2_O_2_ release was calculated using H_2_O_2_ standards and expressed as nmol/1 (Idelman et al., 2015).

### Statistical Analysis

The group size of n = 6 animals for behavioral experiments was determined by sample size estimation using G*Power (v3.1) (Faul et al., 2007) to detect size effect in a post-hoc test with type 1 and 2 error rates of 5 and 20%, respectively. Mice were allocated to vehicle or treatment groups using a randomization procedure (http://www.randomizer.org/). Investigators were blinded to the treatments, which were revealed only after data collection. No animals were excluded from experiments.

Results are expressed as mean ± standard error of the mean (SEM). For multiple comparisons, a one-way analysis of variance (ANOVA) followed by the post-hoc Bonferroni’s test or Dunnett’s test was used. Two groups were compared using Student’s t-test. For behavioral experiments with repeated measures, the two-way mixed model ANOVA followed by the post-hoc Bonferroni’s test was used. Statistical analyses were performed on raw data using Graph Pad Prism 8 (GraphPad Software Inc.). *p* values less than 0.05 (*p* < 0.05) were considered significant. Statistical tests used and the sample size for each analysis are listed in the figure legends.

## Results

### Para-Tibial K7M2 Osteosarcoma Cell Inoculation Induces Mechanical Allodynia and Recruits Macrophages in Tibial Nerve

Para-tibial inoculation of K7M2 osteosarcoma cells into the hindlimb of BALB/c mice induced a time-dependent (0–20 days) increase in hindlimb thickness, mainly due to tumor growth (Figure 1A). Tumor growth was associated with a parallel increase in mechanical allodynia (Figure 1B) and in the number of F4/80^+^/CD64^+^ monocytes/macrophages inside the tibial nerve trunk ipsilateral to the inoculated hindlimb, as shown by flow cytometric analysis and immunofluorescence staining (Figures 1C, D). Control (sham) mice did not develop either the increase in the hindlimb volume or mechanical allodynia or in the number of monocytes/macrophages inside the tibial nerve ipsilateral to the injected hindlimb (Figures 1A–D). These data show a time-dependent association between the tumor mass growth and the development of the mechanical allodynia and for the first time that the tumor growth in K7M2 osteosarcoma-bearing mice is associated with an increased neuroinflammation in the tibial nerve, revealed by the increased number of macrophages (F4/80^+^/CD64^+^ cells).

### β-ARs Antagonism Reduces Osteosarcoma Tumor Growth and Mechanical Allodynia

**FIGURE 2:**
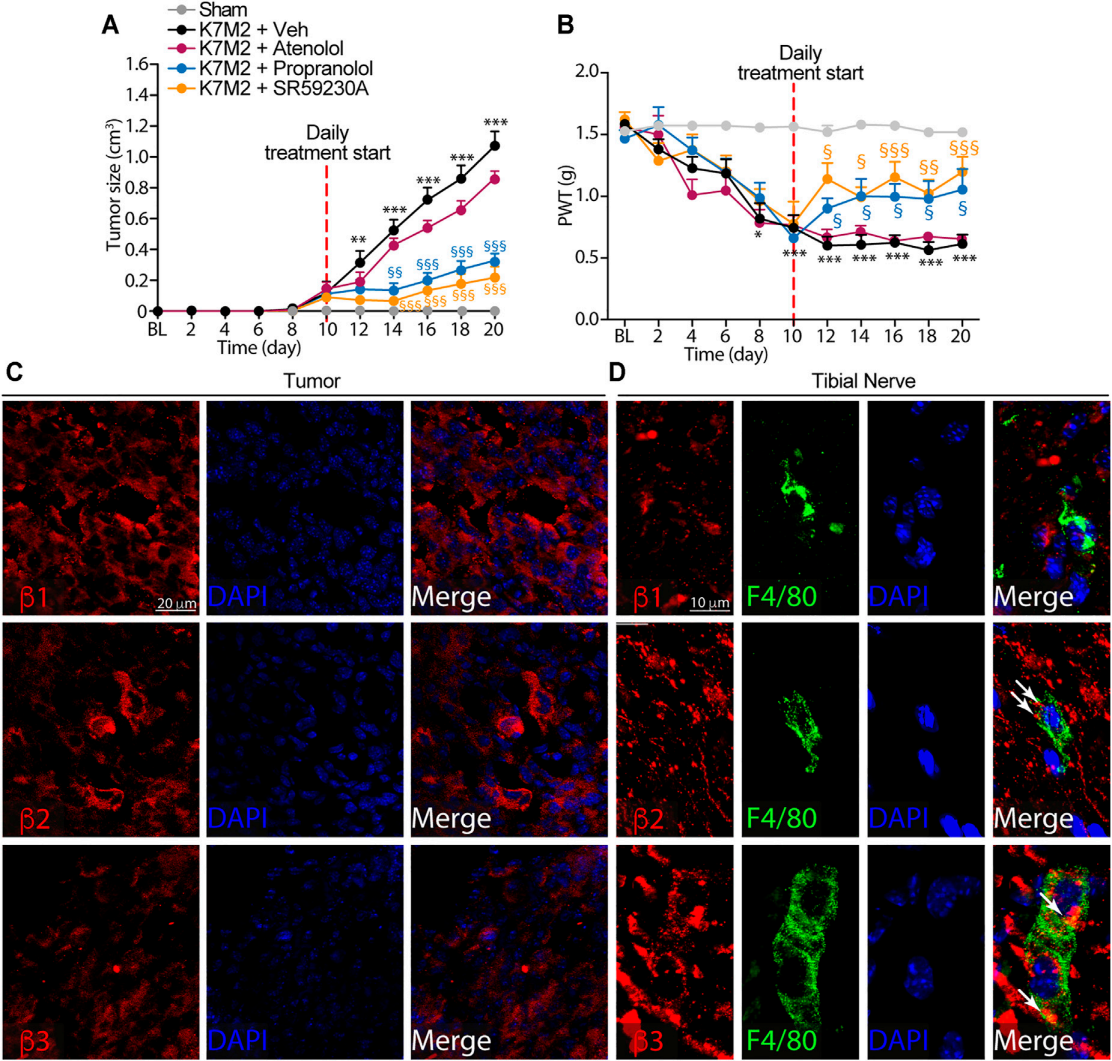
β2-and β3-, but not β1-ARs antagonism reduce K7M2 osteosarcoma growth and mechanical allodynia in BALB/c mice **(A)** Time dependent increase in tumor size and **(B)** mechanical allodynia after para-tibial K7M2 osteosarcoma cell inoculation or sham in BALB/c mice treated (daily, starting from day 10) with atenolol (10 mg/kg, i.p.), propranolol (10 mg/kg, i.p.), SR59230A (10 mg/kg, i.p.), or vehicle (Veh) (C and D) Representative immunofluorescence images of β1-, β2-and β3-ARs expression in **(C)** tumor cells and **(D)** macrophages (F4/80^+^ cells) in tibial nerve, 20 days after para-tibial inoculation of K7M2 osteosarcoma cells. BL, baseline. PWT, paw withdrawal threshold. N = 6 mice. **p < 0.01, ***p < 0.001 vs Sham; ^§^ *p* < 0.05, ^§§^ *p* < 0.01, ^§§§^ *p* < 0.001 vs K7M2 + Veh. Data are presented as mean ± SEM. Two-way ANOVA and Bonferroni post hoc test.

To investigate a possible role of β-ARs in the modulation of tumor growth and mechanical allodynia induced by para-tibial inoculation of K7M2 osteosarcoma cells, 10 days after K7M2 osteosarcoma cells inoculation, when allodynia was already present, mice were treated with the β1-AR antagonist atenolol, the β1-/β2-AR antagonist propranolol, the β3-AR antagonist SR59230A, or vehicle. As already reported in other cancer mouse models (Calvani et al., 2019; Bruno et al., 2020; Calvani et al., 2020), the treatment with β2-and β3-AR antagonists induced a significant reduction in tumor size (Figure 2A), thus confirming a pivotal role of β2-and β3-ARs in tumor growth. Conversely, the treatment with atenolol induced a slight, but not significant, reduction in the tumor size, denoting a minor role of the β1-AR subtype in controlling tumor growth (Figure 2A). Surprisingly, K7M2 osteosarcoma-bearing mice treated with propranolol and SR59230A, but not with atenolol exhibited a marked reduction in the mechanical allodynia (Figure 2B).

To investigate the role played by the β-ARs in tumor and in macrophages accumulating in the neural space, we first evaluated the expression of the three β-AR subtypes in tumor cells and in macrophages located in the tibial nerve. Immunofluorescence staining showed the expression of all three β-AR subtypes in the tumor cells (Figure 2C). Macrophages only expressed β2-and β3-but not β1-AR (Figure 2D).

Overall, these data showed that while β-ARs blockade, mainly β2-and β3-ARs and to a lesser extent β1-AR, is able to significantly influence the osteosarcoma tumor growth, the targeting of β2-and β3-ARs, but not of β1-AR, mitigated the established mechanical allodynia resulting from osteosarcoma tumor growth.

### β2-and β3-ARs Antagonism on Neural Macrophages Modulates Neuroinflammation Responsible of Mechanical Allodynia

We previously observed that the development of mechanical allodynia in several neuropathic pain models (De Logu et al., 2017; De Logu et al., 2020a; De Logu et al., 2020b; De Logu et al., 2021) was associated with the increase in monocytes/macrophages inside the sciatic nerve ipsilateral to the injury. Thus, based on results showing the efficacy of β-AR antagonism to reduce the mechanical allodynia (Figure 2B), we then investigated whether the number of macrophages recruited in tibial nerve of osteosarcoma-bearing mice, could be modulated by β-AR antagonists administration.

**FIGURE 3:**
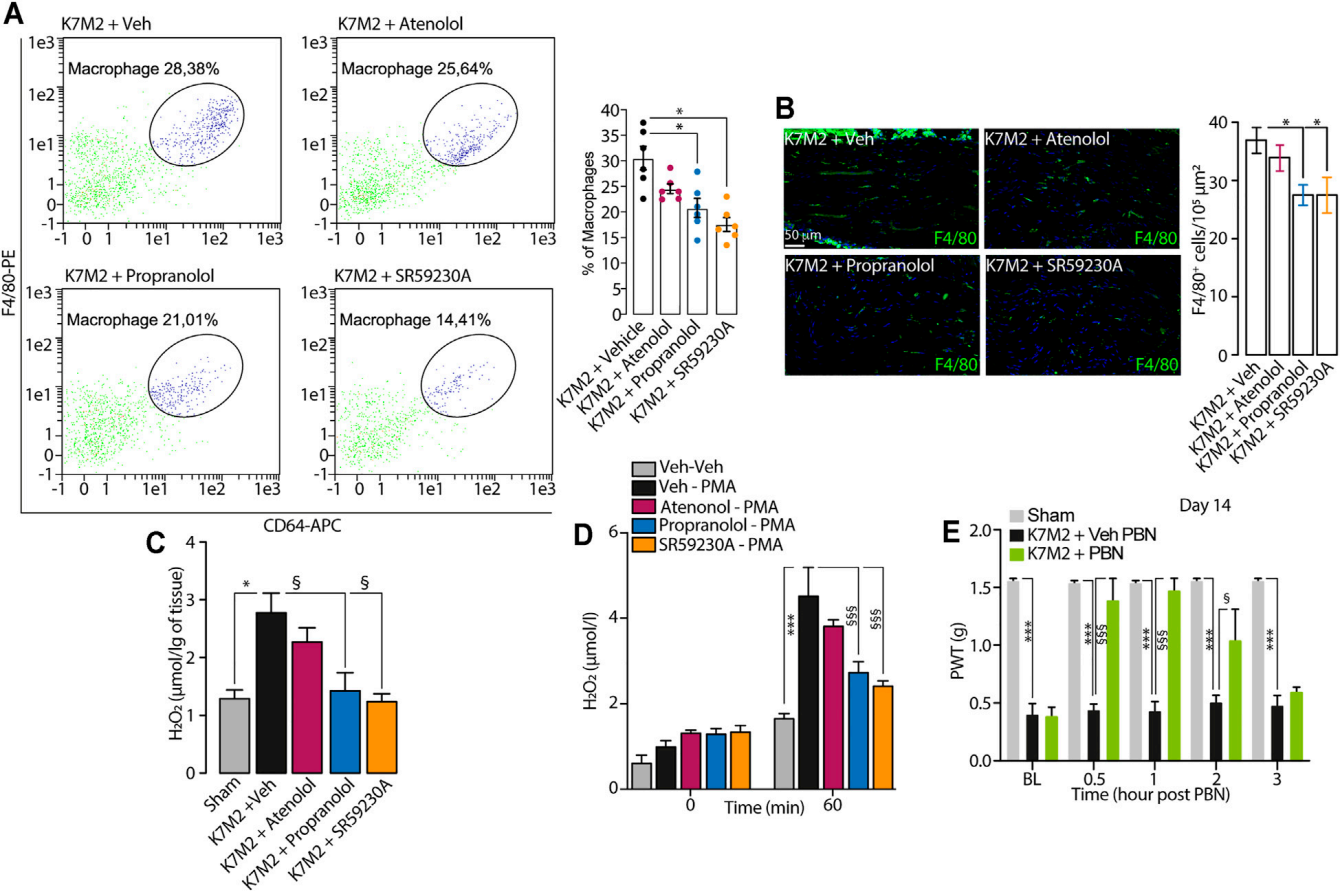
β2-and β3-AR antagonists reduce neuroinflammation in tibial nerve of K7M2 osteosarcoma-bearing mice **(A)** Representative flow cytometry analysis and relative quantification of F4/80^+^/CD64^+^ macrophages **(B)** typical immunofluorescence images and pooled data of F4/80^+^ cells and **(C)** H_2_O_2_ content in the tibial nerve after para-tibial K7M2 osteosarcoma cell inoculation or sham in BALB/c mice treated (daily, starting from day 10) with atenolol (10 mg/kg, i.p.) propranolol (10 mg/kg, i.p.), SR59230A (10 mg/kg, i.p.) or vehicle (Veh) **(D)** H_2_O_2_ level in RAW 264.7 stimulated with PMA (16 μM) in presence of SR59230A, propranolol, atenolol (all, 100 nM) or Veh (n = 3 independent experiments) **(E)** Mechanical allodynia at day 14 after para-tibial K7M2 osteosarcoma cell inoculation or sham in BALB/c mice treated with PBN (100 mg/kg, i.p.) or Veh. BL, baseline. PWT, paw withdrawal threshold. N = 6 mice. *p < 0.05, ***p < 0.001 vs Sham, Veh-Veh; ^§^ *p* < 0.05, ^§§§^ *p* < 0.001 vs K7M2 + Veh, Veh-PMA, K7M2 + Veh PBN. Data are presented as mean ± SEM. One-way (A–C) or two-way (D–E) ANOVA and Bonferroni post hoc test.

We observed that the repeated treatment with the β1/2- and β3-AR antagonists (propranolol and SR59230A), but not with the β1-AR antagonist (atenolol), showed a significant reduction in the number of macrophages in tibial nerve compared to vehicle-treated mice (Figures 3A, B).

It has been known that the increase in macrophages number evokes an increase in oxidative stress at the tissue level, leading to the development of pain (De Logu et al., 2017; De Logu et al., 2020a; De Logu et al., 2020b; De Logu et al., 2021). Accordingly, in the tibial nerve of K7M2 osteosarcoma-bearing mice, we observed an increase in H_2_O_2_ levels compared to sham mice (Figure 3C), and the repeated treatment with propranolol and SR59230A, but not with atenolol, significantly reduced the increase in H_2_O_2_ levels in tibial nerve (Figure 3C). Given the absence of the β1-AR expression in macrophages, we speculated that the analgesic effect observed in K7M2 osteosarcoma-bearing mice following the repeated treatment with propranolol and SR59230A, was mainly due to a modulation of neuroinflammation (H_2_O_2_ release) dependent on β2-and β3-ARs blockade on tibial macrophages. To corroborate our hypothesis, we stimulated a murine macrophage cell line (RAW 264.7) with the pro-oxidant agent phorbol 12-myristate 13-acetate (PMA) alone or in combination with the different β-AR antagonists. Results showed that PMA induced a release of H_2_O_2_ from RAW 264.7 macrophages which was reduced in the presence of β2-and β3-AR but not with β1-AR antagonists (Figure 3D).

To finally confirm that the increase in H_2_O_2_ level was associated to mechanical allodynia in our model, K7M2 osteosarcoma-bearing mice were treated with the antioxidant PBN, at day 14 after cells inoculation, when mechanical allodynia was already established. Data showed that a single injection of PBN was able to transiently revert mechanical allodynia (Figure 3E).

Overall, our data suggest that macrophages accumulating in tibial nerve of K7M2 osteosarcoma-bearing mice induce the development of mechanical allodynia by releasing oxidative stress, and that this process is finely regulated by β2-and β3-but not by β1-ARs modulation.

## Discussion

Cancer-induced pain represents a high-priority complication related to tumor growth, not only in primary bone cancer, but also in bone cancer metastasis. Despite the growing interest in the research of new treatments for cancer pain, opioids remain the leading therapeutic option, which, however, shows marked limitations. Poor understanding of molecular mechanisms implicated in the development and maintenance of cancer-induced pain limits the identification of novel therapeutic targets for this condition. Previous studies highlighted the role of β-ARs modulation in models of various neuropathic pain (Kanno et al., 2010; Fávaro-Moreira et al., 2012; Zhang et al., 2018). However, the mechanism by which these receptors promote the development and maintenance of pain is poorly understood. Our data show that β-ARs, mainly β2-and β3-ARs sustain both tumor growth and cancer pain in a syngeneic osteosarcoma murine model. Indeed, the systemic treatment with the β1-AR antagonist atenolol did not affect osteosarcoma tumor growth, while the β1/2- and β3-ARs antagonists, propranolol and SR59230A respectively, reduced osteosarcoma tumor growth and were also able to modulate the mechanical allodynia developed with the tumor growth. As these receptors are expressed both on tumor cells and inflammatory cells, including macrophages, administration of β-ARs antagonists is able to target both of these pathological processes.

First, we showed that the osteosarcoma-bearing mice developed a sustained mechanical allodynia, starting from day 10 after K7M2 osteosarcoma cells inoculation, that was absent in sham mice. Then, based on previous data that showed the pivotal role of peripheral nerve macrophages in the development of pain in mouse models of neuropathic and cancer pain (De Logu et al., 2017; De Logu et al., 2020a; De Logu et al., 2021), we wondered whether also in this cancer model, macrophages in the peripheral (tibial) nerve, which runs ipsilateral to the tumor, played a role in the development and maintenance of mechanical allodynia. Data showed that the number of neural macrophages in the tibial nerve of osteosarcoma-bearing mice were clearly increased compared to sham mice.

To test the involvement of the β-ARs in the maintenance of cancer-related pain in osteosarcoma-bearing mice, we treated the mice (daily) with the β1-AR antagonist atenolol, the β1/2-AR antagonist propranolol and the β3-AR antagonist SR59230A. Results showed that atenolol treatment had no effect in reducing mechanical allodynia, while propranolol and SR59230A treatment significantly reduced the mechanical allodynia. Moreover, propranolol and SR59230A were also able to reduce the tumor growth, confirming the crucial role of the β2-and β3-AR subtypes in sustaining pro-tumoral signaling in agreement with previous data obtained in other mouse models of cancer (Lamkin et al., 2012; Magnon et al., 2013; Calvani et al., 2019; Bruno et al., 2020; Calvani et al., 2020).

Since the development of mechanical allodynia was associated with an increase in neural macrophages and that β-ARs antagonism was able to reduce osteosarcoma-induced pain, we wondered if there was a correlation between β-ARs activity and the modulation of neuroinflammation. Surprisingly, β2-and β3-ARs antagonists were able to significantly reduce the number of macrophages in the tibial nerve compared to vehicle treated mice. According to previously reported data showing that peripheral β2-and β3-ARs drive functional pain *via* increased activation of immune cells which promote neuroinflammation and neuropathic pain (Kanno et al., 2010; Zhang et al., 2018), and in the light of our previous data (De Logu et al., 2017; De Logu et al., 2020a; De Logu et al., 2021), our results suggested that the reduction in mechanical allodynia observed in propranolol- and SR59230A-treated mice was partially due to the reduced inflammatory cells (macrophages) in tibial nerve associated with the decreased tumor size following drug treatment. Furthermore, the evaluation of β-ARs expression in osteosarcoma tumor and in neural (tibial nerve) macrophages, showed that while all the three β-ARs were expressed in the tumor, only the β2-and β3-AR subtypes were expressed on neural macrophages. These results, together with data showing that both β1/2-AR and β3-AR but not β1-AR antagonism was able to affect cancer-evoked mechanical allodynia, supported the hypothesis that β2-and β3-AR signaling on neural macrophages sustained the mechanical allodynia developed in osteosarcoma-bearing mice.

To corroborate this hypothesis, we focused our attention to the increase in the oxidative stress as biological process correlated to the macrophages activity in the tibial nerves, and responsible for the nociceptor activation and the ensuing pain condition (De Logu et al., 2017). We observed that, in tibial nerves of osteosarcoma-bearing mice, the amount of H_2_O_2_, a reactive oxygen species (ROS) known to be responsible of nociception activation in several pain model (Antoniazzi et al., 2019; Brusco et al., 2020; De Logu et al., 2021), resulted increased compared to sham mice, and that treatment with propranolol and SR59230A, but not with atenolol, abrogated this effect *in vivo*. Accordingly, ROS (H_2_O_2_) production in murine macrophages was reverted by the treatment with propranolol and SR59230A, but not with atenolol. Finally, we reported that the treatment with an antioxidant transiently abrogated the established mechanical allodynia *in vivo*, thus confirming a leading role of oxidative burst in maintaining pain condition.

To date, there are no clinical studies aiming at investigating whether the use of β-AR antagonists could be effective in counteract cancer-pain in humans. However, a phase 2 study (NCT01222091) aimed at exploring whether propranolol treatment improved thermal and mechanical hypersensitivity after administration of an opioid (remifentanil), showed that concomitant infusion of propranolol with remifentanil prevented the remifentanil-induced post infusion hyperalgesia (Chu et al., 2012). These data highlight the great significance of investigating the role of the β-adrenergic system as pharmacological target for preventing different type of pain.

Overall, our data suggest a role for the β-ARs signaling, mainly through β2-and β3-ARs, in sustaining both tumor growth and cancer-induced pain in a syngeneic osteosarcoma murine model and identify macrophages recruited in the peripheral nerve and oxidative stress generation, as cellular and molecular mediators for the development of such pain. Furthermore, our study highlights the ability of β-ARs antagonists in modulating both tumor growth and the induction of cancer-evoked pain, giving to these receptors unique features as promising therapeutic targets for cancer therapy and associated pain.

## Data Availability

The raw data supporting the conclusions of this article will be made available by the authors, without undue reservation.
